# The Effects of Clazakizumab on Peripheral Blood and Kidney Transcriptomes in Patients With Late Antibody-Mediated Rejection

**DOI:** 10.1016/j.ekir.2025.08.027

**Published:** 2025-08-26

**Authors:** Roy Zhang, Colin Y.C. Lee, Martina Schatzl, Klemens Budde, Fabian Halleck, Bernd Jilma, Jessica Chang, Philip Halloran, Georg A. Böhmig, Menna R. Clatworthy

**Affiliations:** 1 Molecular Immunity Unit, Department of Medicine, University of Cambridge, Cambridge, UK; 2 Cambridge Institute for Therapeutic Immunology and Infectious Diseases, Cambridge, UK; 3 Cellular Genetics, Wellcome Sanger Institute, Hinxton, UK; 4 Division of Nephrology and Dialysis, Department of Medicine III, Medical University of Vienna, Vienna, Austria; 5 Department of Nephrology, Charité Universitätsmedizin Berlin, Berlin, Germany; 6 Department of Clinical Pharmacology, Medical University of Vienna, Vienna, Austria; 7 Alberta Transplant Applied Genomics Centre, University of Alberta, Edmonton, Alberta, Canada

**Keywords:** antibody-mediated rejection, clazakizumab, interleukin-6 (IL-6), transcriptomics

## Abstract

**Introduction:**

There are no licensed treatments for antibody-mediated rejection (AMR), a major cause of late kidney allograft loss. Clazakizumab (CLZ), an interleukin (IL)-6–neutralizing antibody, showed potential efficacy in a phase 2 trial in late AMR, with a reduction in donor-specific antibodies (DSAs) and kidney molecular microscope diagnostic system (MMDx) AMR score, but the underpinning mechanisms are unclear.

**Methods:**

Using peripheral blood transcriptomics, we identified a decrease in IL-6–associated “JAK-STAT signaling” pathway genes with CLZ, and a reduction in gene modules that enriched for T follicular helper cell and activated platelet signatures, cells that contribute to DSA generation and inflammatory responses to DSA respectively. However, responses were variable, and some patients showed a rebound in the expression of inflammatory signatures with long-term CLZ treatment, indicating variability in the efficacy of IL-6 antagonism. One peripheral blood gene module significantly correlated with kidney MMDx AMR score and enriched for monocyte signature genes, as well as “Fc gamma receptor–mediated phagocytosis” and “leukocyte transendothelial migration” gene sets, suggesting that cells activated by DSAs can be detected in peripheral blood. In the kidney, CLZ-treatment was associated with a significant reduction in a damaged tubule gene signature and preservation of podocyte signatures. We also found a kidney plasma cell gene–rich module that positively correlated with circulating DSAs; however, this was not significantly downregulated by CLZ.

**Conclusion:**

Overall, our results provide mechanistic insights into the effects and limitations, of IL-6 neutralization in humans in the context of AMR.

## Graphical abstract

**Figure:**
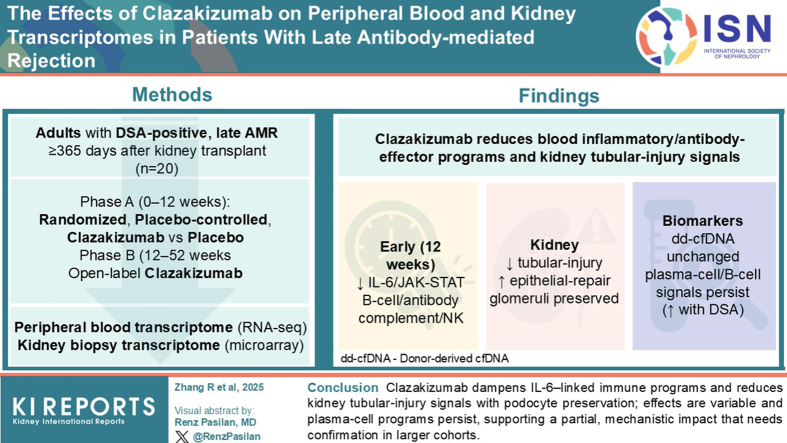

See Commentary on Page 3741

Late AMR is a significant cause of allograft loss following kidney transplantation.^1^^,^^2^ Despite the increased understanding of its pathogenesis and molecular basis,^3^^,^^4^ proven, effective therapeutic options are not well-established.^5^ Attempts to reduce antibody-producing cells with bortezomib^6^ or rituximab combined with iv. Ig,^7^ have not shown significant effects on relevant clinical, serological, morphological, or molecular parameters.

IL-6 is a pleotropic cytokine that promotes the survival and maturation of B cells and plasmablasts, is required for CD4 T-cell polarization to T follicular helper cells (Tfh),^8^ and has additional effects on innate immunity, increasing monocyte differentiation to macrophages^9^ and platelet production and activation.^10^ IL-6 has been strongly implicated in AMR^11^; IL-6 receptor blockade attenuated alloantibody responses in mice^12^ and was associated with a reduction in DSAs and stabilization of graft function in patients with late AMR.^13^ CLZ, an IL-6–neutralizing humanized monoclonal IgG1 antibody, was assessed in a phase 2 randomized controlled trial for late active AMR, where patients were randomized to receive either placebo (PBO) or CLZ for 12 weeks, followed by CLZ in both groups for 40 weeks.^14^ Prolonged CLZ treatment for 12 months led to a reduction in DSAs, slowing of estimated glomerular filtration rate decline, and lower kidney biopsy MMDx AMR score, highlighting its therapeutic potential.^14^ However, the molecular mechanisms underpinning this effect have not been addressed, including whether its action in AMR is because of direct effects of IL-6 neutralization on B-cell activation and inhibition of Tfh differentiation, which together may reduce DSAs, or additional potential effects on platelets (known players in AMR^15^^,^^16^) or monocyte or macrophage activation within the graft. This initial promise led to the use of CLZ in a larger, phase 3 clinical trial in late AMR, IMAGINE^17^ (https://clinicaltrials.gov/study/NCT03744910); however, this study has recently been terminated early because of lack of efficacy in the interim analysis.^18^ Molecular insights into why IL-6 neutralization in isolation may not be fully efficacious would be helpful for the community to rationalize future therapeutic strategies in this area of need.

Although transcriptomic analyses have been applied to kidney biopsies to identify gene signatures for AMR,^19^^,^^20^ a peripheral blood signature that accurately reflects AMR within the graft has been elusive, but could provide a noninvasive means of monitoring and assessing treatment efficacy, including in the context of clinical trials.^21^, ^22^, ^23^ Notably, studies with paired transcriptional data from blood and kidney, that might provide a more sensitive assessment of processes in the kidney, compared with histological changes in biopsy morphology, are lacking.

Here, we assessed transcriptional changes in peripheral blood in patients enrolled in the CLZ in late AMR phase 2 study,^14^ integrating this with the kidney microarray data generated as part of the original trial MMDx biopsy end points. We aimed to investigate the effects of IL-6 blockade on circulating immune cells, the extent to which these reflected molecular changes in the allograft, and whether we could gain insights into why CLZ treatment may not show full efficacy in AMR.

## Methods

### Trial Design and Ethics

**Figure 1:**
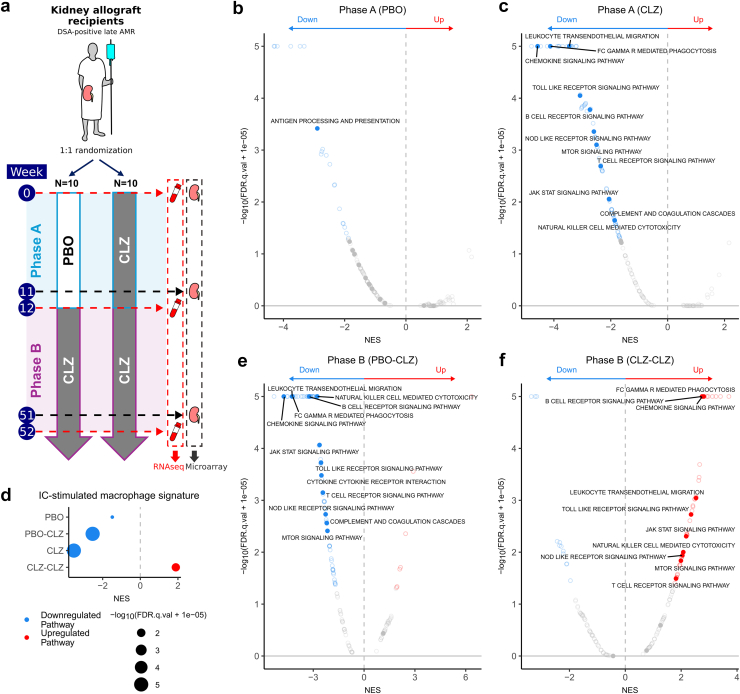
Effects of clazakizumab on the peripheral blood transcriptome in late antibody-mediated rejection (AMR). Peripheral blood samples were taken at week 0, week 12, and week 52 in a clazakizumab in late AMR study and analyzed by RNA sequencing (RNA-seq). CLZ, clazakizumab; DSA, donor-specific antigen; GSEA, gene set enrichment analysis; NES, normalized enrichment score; PBO, placebo. (a) Summary schematic of experimental design. *n* = 20 participants with DSA-positive late AMR, with estimated glomerular filtration rate > 30 ml/min per 1.73 m^2^ and ≥ 365 days posttransplantation, were randomized to receive either placebo or clazakizumab monthly for 12 weeks (phase A) followed by clazakizumab (phase B). Peripheral blood samples were taken at baseline (week 0), following phase A (week 12) and following phase B (week 52). (b) GSEA of the differential expression analysis of week 12 versus week 0 samples in the placebo arm, using the KEGG pathways. Red dots indicate pathways upregulated during phase A in the placebo arm; blue indicates downregulated pathways. Selected immune pathways are solid dots and labelled when significant (FDR *q*-value < 0.05). (c) GSEA of the differential expression analysis of week 12 versus week 0 samples in the clazakizumab arm, using the KEGG pathways. Red dots indicate pathways upregulated during phase A in the clazakizumab arm; blue indicates downregulated pathways. Selected immune pathways are solid dots and labelled when significant (FDR *q*-value < 0.05). (d) GSEA of the differential expression analyses across phase A and phase B for both study arms, using an immune complex–stimulated macrophage signature.^25^ Only significant pathways (FDR *q*-value < 0.05) are plotted. Red dots indicate positive enrichment across the trial phase and blue negative; the size of the dot is inversely correlated with the FDR *q*-value and the position indicates the NES. (e) GSEA of the differential expression analysis of week 52 versus week 12 samples in the placebo arm, using the KEGG pathways. Red dots indicate pathways upregulated during phase B in the placebo arm; blue indicates downregulated pathways. Selected immune pathways are solid dots and labelled when significant (FDR *q*-value < 0.05). (f) GSEA of the differential expression analysis of week 52 versus week 12 samples in the clazakizumab arm, using the KEGG pathways. Red dots indicate pathways upregulated during phase B in the clazakizumab arm; blue indicates downregulated pathways. Selected immune pathways are solid dots and labelled when significant (FDR *q*-value < 0.05).

The design and ethical approval of the clinical phase 2 pilot trial is previously described.^14^^,^^24^ In brief, the study consisted of 2 parts: a 12-week randomized PBO-controlled phase (phase A) followed by a 40-week open-label extension, where all participants received CLZ (phase B). Twenty participants had DSA-positive late AMR at least 365 days posttransplantation. Peripheral blood samples were taken at baseline, 12 weeks, and 52 weeks, while kidney biopsies were taken at baseline, 11 weeks, and 51 weeks (Figure 1a).^25^ Two participants were withdrawn from the study in phase B. Baseline characteristics of the study groups and processing of kidney samples were previously described,^14^ whereas sample metadata is available in Supplementary Table S1.

### RNA Extraction and Sequencing

RNA in whole blood was stabilized and subsequently extracted using a blood RNA kit (PAXgene). RNA concentration was assessed using a nanodrop spectrophotometer (Thermo Scientific) and RNA quality using a Nano Bioanalyzer kit (Agilent) and Bioanalyzer 2100 (Agilent). We used 0.3–0.75 μg of RNA to produce sequencing libraries using TruSeq Stranded total RNA library prep kit (Illumina) with 14 polymerase chain reaction amplification cycles. Libraries were sequenced on a NovaSeq sequencer (Ilumina) by Genewiz.

### RNA-Sequencing Analysis

Sequencing data was demultiplexed to give individual fastq files using Casava (Illumina). FastQC was used for quality control. Fastq files were aligned to the human genome (Hg38) using STAR^26^ and quantified using featureCounts within Rsubread.^27^ Normalization and differential gene expression analysis was carried out using DESeq2 (V1.34.0).^28^ Fifty-six peripheral blood samples were included in the analysis following quality control. Individual and batch effects were included in modelling.

### Microarray Analysis

Microarray data derived from paired kidney samples were obtained as described in Doberer *et al.*^14^ RMA transformation was applied before weighted gene coexpression network analysis (WGCNA). Fifty-six samples were included in the analysis (1 outlier and 1 medullary sample removed, as previous described^29^). Differential gene expression analysis was undertaken in limma.^30^

### WGCNA

WGCNA was performed using the WGCNA package (V1.70-3).^31^ Batch effects were removed using removeBatchEffect function in limma.^30^ Correlation coefficients were calculated between all genes using biweight midcorrelation. This was transformed into an adjacency matrix using soft thresholds, approximating scale-free topology. A hybrid dynamic tree cut algorithm was applied on the dissimilarity topological overlap matrix to obtain coexpression modules. Highly correlated modules were merged. Module membership (correlation between gene expression and module eigengene) > 0.8 was used to define hub genes. Calculation of module preservation statistics is detailed in the Supplementary Methods.

### Pathway Analysis

For gene set enrichment analysis (GSEA), we used MSigDB GSEA software (V4.1.0), with genes ranked by the inverse of the *P*-value with the sign of the log-fold change^32^ or module membership in WGCNA. Signatures used are described in the Supplementary Methods. Gene ontology analysis was performed using topGO (v2.46.0).^33^ The gene universe was hub genes of any module. Single sample GSEA (ssGSEA) was performed on GenePattern (https://genepattern.org) using the ssGSEA module (v10.1.0).^32^^,^^34^

### STRING Analysis

Functional gene interaction networks were constructed using the STRING database (https://string-db.org/).^35^

## Results

### Short-Term Reduction in Antibody Effector Gene Signatures in Peripheral Blood Following CLZ

Peripheral blood samples were available at baseline, 12 weeks, and 52 weeks in patients recruited to a phase 2 randomized trial of CLZ in late AMR (Figure 1a). Fifty-six peripheral blood samples were included in analysis. During phase A, (12-week treatment with CLZ vs. PBO), several immune pathways were significantly downregulated at week 12 compared with pretreatment in the CLZ arm. This included gene sets relating to the known effects of IL-6, for example, “JAK-STAT signaling” and “B cell receptor signaling,” and gene sets reflecting important antibody-effector pathways, such as “FcγR-mediated phagocytosis,” “complement and coagulation cascades” and “natural killer (NK) cell–mediated cytotoxicity.” with no downregulation of these pathways observed in the PBO group (Figure 1b and c). Leading edge genes included *FCGR2A*, *FCGR3A*, and *FCGR3B*, as well as molecules involved in signaling downstream of FcγRs (Supplementary Table S2 and S3). Downregulation of immune “Hallmarks” gene sets, including “TNFA via NFKB” and “IL-6 JAK STAT3 signaling,” were more prominent in the CLZ compared with PBO (Supplementary Figure S1a and b) and we observed a marked reduction in the expression of a reference gene signature generated from IgG immune complex–stimulated macrophages^25^ in the CLZ group (Figure 1d). This reduction of antibody effector and FcγR-mediated activation-associated gene sets in peripheral blood is somewhat surprising, because FcγR ligation requires IgG immune complexes or tissue-deposited IgG, and cannot be triggered by circulating monomeric IgG,^36^ suggesting that some of the transcriptional changes detectable in blood may reflect a change in tissue activation of FcγR-expressing monocytes or NK cells by DSA.

During phase B (weeks 12–52), all patients received CLZ (Figure 1a). In patients switched from PBO to CLZ (PBO-CLZ), similar pathways were downregulated as in the phase A CLZ-treated patient samples, with antibody-effector pathways among the most negatively enriched (Figure 1e, Supplementary Figure S1C). Surprisingly, when considering all patients continuing on CLZ during phase B (CLZ-CLZ), there was a significant upregulation of some immune pathways (Figure 1f, Supplementary Figure S1D), including genes expressed by IgG immune complex–stimulated macrophages^25^ (Figure 1d).

### Variable Responses to CLZ With Some Patients Showing a Rebound in Inflammatory Gene Sets With Long-Term Treatment

**Figure 2:**
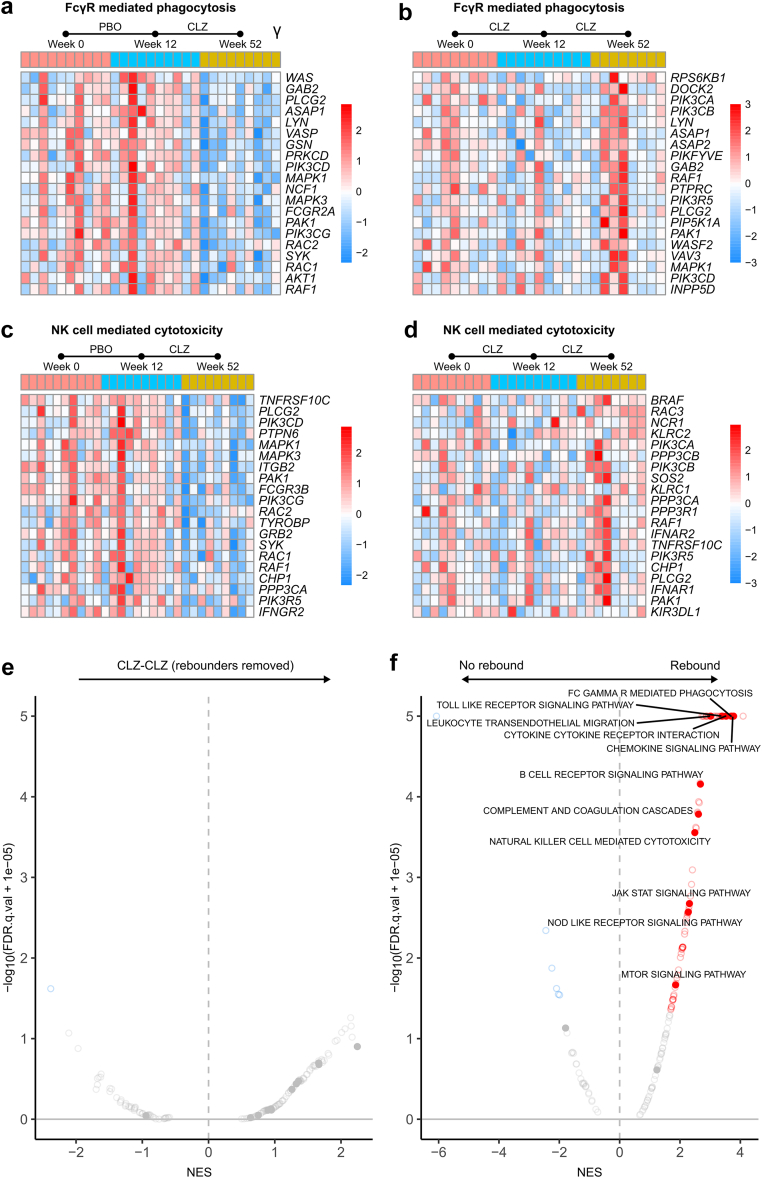
Leading edge gene analysis shows variable treatment effect with possible rebound in certain individuals. Peripheral blood samples were taken at week 0, week 12, and week 52 in a clazakizumab in late antibody-mediated rejection (AMR) study and analyzed by RNA sequencing (RNA-seq). CLZ, clazakizumab; GSEA, gene set enrichment analysis; NES, normalized enrichment score; PBO, placebo. (a) Heatmap of top 20 leading edge genes for the GSEA of “FcγR mediated phagocytosis pathway” in the placebo arm. Samples (columns) have been classified by the sample time point. Expression data has been variance-stabilized and scaled by row (color represents Z-score). (b) Heatmap of top 20 leading edge genes for the GSEA of “FcγR mediated phagocytosis pathway” in the clazakizumab arm. Samples (columns) have been classified by the sample time point. Expression data has been variance-stabilized and scaled by row (color represents Z-score). (c) Heatmap of top 20 leading edge genes for the GSEA of “natural killer (NK) cell–mediated cytotoxicity” in the placebo arm. Samples (columns) have been classified by the sample time point. Expression data has been variance-stabilized and scaled by row (color represents Z-score). (d) Heatmap of top 20 leading edge genes for the GSEA of “natural killer (NK) cell–mediated cytotoxicity” in the clazakizumab arm. Samples (columns) have been classified by the sample time point. Expression data has been variance-stabilized and scaled by row (color represents Z-score). (e) GSEA of the differential expression analysis of week 52 versus week 12 samples in the clazakizumab arm, using the KEGG pathways, following exclusion of the rebounding samples. Red dots indicate pathways upregulated during phase B in the clazakizumab arm; blue indicates downregulated pathways. Selected immune pathways are solid dots and labelled when significant (FDR *q*-value < 0.05). (f) GSEA of the differential expression analysis of week 0 samples of individuals who had rebound with long-term clazakizumab treatment versus week 0 samples of individuals who did not, using the KEGG pathways. Red dots indicate pathways upregulated in samples at week 0 who will have rebound following long-term clazakizumab treatment; blue indicates downregulated pathways. Selected immune pathways are solid dots and labelled when significant (FDR *q*-value < 0.05).

Closer inspection of the leading-edge genes in each patient within treatment groups revealed variable individual patient responses to treatment (Figure 2a–d). Of note, this within-group variation was less evident in the PBO-CLZ group following 36 weeks of CLZ compared with 12 weeks CLZ in phase A. Furthermore, the rebound increase in immune gene set expression in phase B of the CLZ-CLZ group was largely driven by increased inflammatory gene expression in 3 patients (Figure 2b and d). These individuals showed less suppression of antibody effector gene sets at the 12-week time point, suggesting individual variability in responses to CLZ, and the development of a refractory state to IL-6 neutralization in some patients. With the exclusion of these samples from the analysis, significant upregulation of immune pathways was no longer present (Figure 2e). Although these patients were not distinguished by reduced renal function, increased DSAs at the time of sampling or enrichment of immune pathways in the kidney (Supplementary Figure S2A–D), it is unclear if they would have accelerated graft failure over longer follow-up. Of note, these individuals also had increased enrichment of immune pathways compared with pretreatment samples from those who did not have rebound in phase B (Figure 2f), suggesting that blood transcriptomics could be used to identify patients that may require additional or more intensive therapy. These patients were not distinguishable pretreatment using more conventional means of assessment such as serological or histological assessment, although sample size was limited (Supplementary Table S4). This variability in response may have led to a lack of efficacy in the larger, phase 3 clinical trial of CLZ in late AMR, which was terminated early.

### WGCNA Links a Treatment-Associated Monocyte Gene-Rich Module in Peripheral Blood With MMDx AMR Score

**Figure 3:**
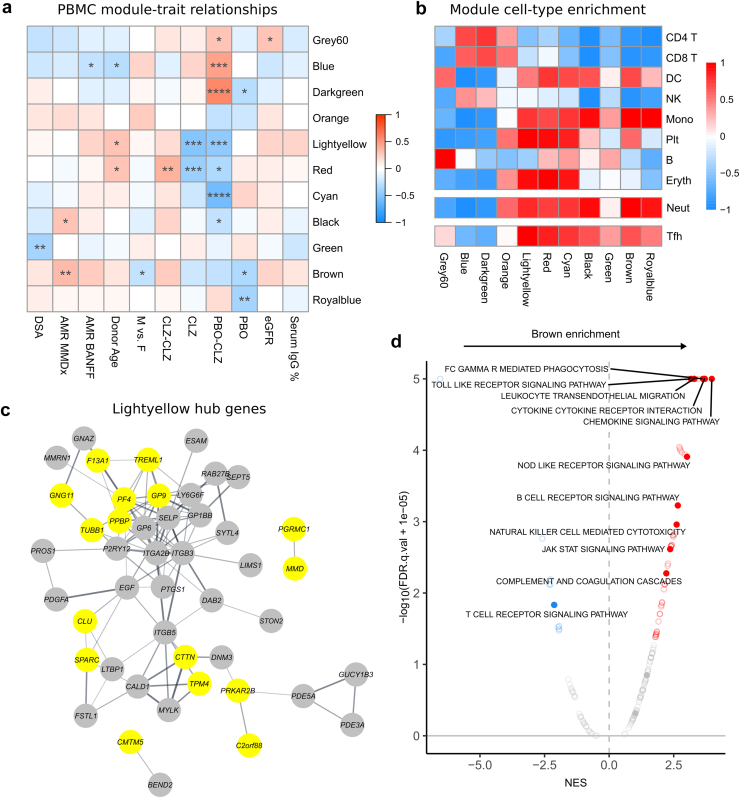
Weighted gene coexpression network analysis (WGCNA) of peripheral blood samples. Peripheral blood samples were taken at week 0, week 12, and week 52 in a clazakizumab group in late AMR study and underwent RNA sequencing (RNA-seq). (a) Module-trait relationships for PBMC samples between identified modules (rows) and clinical parameters of interest (columns). Heatmap colors indicate correlation between module eigengene and clinical parameter. Asterisks indicate *P*-values of correlations (∗, ≤ 0.05; ∗∗, ≤ 0.01; ∗∗∗, ≤ 0.001; ∗∗∗∗, ≤ 0.0001). AMR, antibody-mediated rejection; CLZ, Clazakizumab group phase A; CLZ-CLZ, clazakizumab group phase B; DSA, donor-specific antibody; eGFR, estimated glomerular filtration rate; MMDx, molecular microscope diagnostic test (see Methods); PBMC, peripheral blood mononuclear cells; PBO, placebo group phase A; PBO-CLZ, placebo group phase B. (b) Heatmap showing enrichment of cell types in modules identified with WGCNA. Enrichment scores were calculated using single-sample gene set enrichment analysis (ssGSEA) and scaled by row. Red indicates positive enrichment; blue indicates negative. Rows are split into the 4 datasets from which signatures were derived.^37^, ^38^, ^39^ DC, dendritic cell; Eryth, erythrocyte; Mono, monocyte; Neut, neutrophil; NK, natural killer cell; Plt, platelet; Tfh, T follicular helper cell. (c) STRING analysis of hub genes of the Lightyellow module identified with WGCNA. Hub genes were defined as those with module membership > 0.8. Only connected nodes are displayed. Edges represent confidence score > 0.4 between genes; thicker line = stronger connection. (d) GSEA of the Brown module identified with WGCNA. Genes were ranked by module membership. Red dots indicate pathways positively enriched in the Brown module; blue indicates negative enrichment. Selected immune pathways are solid dots and labelled when significant (FDR *q*-value < 0.05).

To further assess transcriptional changes associated with traits such as treatment, DSA levels, and kidney biopsy findings, we used WGCNA. This identified 11 eigengene modules that were allocated color labels by convention (Supplementary Figure S3A). Broadly, there were concordant effects of shorter-term CLZ treatment in both study groups, with decreased expression of Lightyellow, Red, and Cyan modules in both the CLZ-CLZ phase A and PBO-CLZ phase B samples, defining the major effects of CLZ on peripheral blood immune cells (Figure 3a).^37^, ^38^, ^39^ Cellular deconvolution revealed that monocyte, neutrophil and platelet-specific genes were enriched across these modules (Figure 3b). The Lightyellow module included many platelet-specific hub genes (Figure 3c, Supplementary Table S5), the latter consistent with the known effects of IL-6 on thombompoesis,^40^ platelet activation,^10^^,^^41^ and in stimulating platelet-activating factor production by myeloid cells.^42^ The Lightyellow module also enriched for Tfh signature genes (Figure 3b, Supplementary Figure S3B).

The brown module was downregulated in the CLZ-CLZ phase A and PBO-CLZ phase B samples (although this did not reach statistical significance) (Figure 3a). This module was the one that most enriched for monocyte and neutrophil signature genes, and interestingly, showed a significant positive correlation with a higher MMDx AMR score in the kidney^14^^,^^19^^,^^20^ (*P* = 0.008; Figure 3a). It also enriched for immune gene sets including “leucocyte transendothelial migration,” “chemokine signaling pathway” and “Fc gamma receptor–mediated phagocytosis” (Figure 3d). Enriched gene ontology terms included “mononuclear cell migration,” and hub genes included *S100A8* and *S100A9* that form calprotectin, a neutrophil cytoplasmic protein,^43^ and *FCGR2A* and *FCGR3B*,^44^ surface receptors expressed by monocytes and neutrophils respectively (Supplementary Tables S6 and S7). This raises the possibility that myeloid cell activation by DSAs within the kidney may be detectable in peripheral blood.

### CLZ-Treatment Associated With a Reduction in “Damaged” Tubular Signatures in the Kidney

**Figure 4:**
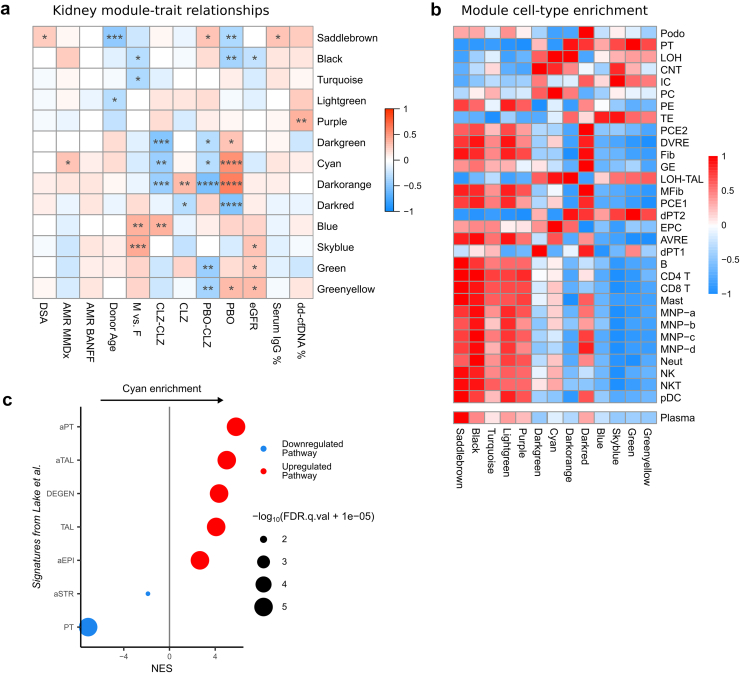
Weighted gene coexpression network analysis (WGCNA) of kidney biopsy samples. Kidney biopsies were taken alongside peripheral blood samples at week 0, week 11, and week 51 in a clazakizumab in late AMR study and analyzed by microarray. (a) Module-trait relationships for kidney samples between identified modules (rows) and clinical parameters of interest (columns). Heatmap colors indicate correlation between module eigengene and clinical parameter. Asterisks indicate *P*-values of correlations (∗, ≤ 0.05; ∗∗, ≤ 0.01; ∗∗∗, ≤ 0.001; ∗∗∗∗, ≤ 0.0001). AMR, antibody-mediated rejection; CLZ, clazakizumab group phase A; CLZ-CLZ, clazakizumab group phase B; dd-cfDNA, donor-derived cell-free DNA; DSA, donor-specific antibody; eGFR, estimated glomerular filtration rate; MMDx, molecular microscope diagnostic test (see Methods); PBMC, peripheral blood mononuclear cells; PBO, placebo group phase A; PBO-CLZ, placebo group phase B. (b) Heatmap showing enrichment of cell types in modules identified with WGCNA. Enrichment scores were calculated using single-sample gene set enrichment analysis (ssGSEA) and scaled by row. Red indicates positive enrichment; blue indicates negative. Rows are split into the 2 datasets from which signatures were derived.^45^^,^^46^ AVRE, ascending vasa recta endothelium; CNT, connecting tubule; dPT, distinct proximal tubule; DVRE, descending vasa recta endothelium; EPC, epithelial progenitor cell; Fib, fibroblast; GE, glomerular endothelium; IC, intercalated cell; LOH, loop of Henle; LOH-TAL, loop of Henle thick ascending limb; MFib, myofibroblast; MNP, mononuclear phagocyte; Neut, neutrophil; NK, natural killer cell; NKT, natural killer T cell; PC, principal cell; PCE, peritubular capillary endothelium; pDC, plasmacytoid dendritic cell; PE, pelvic epithelium; Podo, podocyte; PT, proximal tubule; TE, transitional epithelium. (c) Gene set enrichment analysis (GSEA) of Cyan module against signatures from Lake et al.^47^ Module membership was used to rank all genes. Only significant gene sets (FDR *q*-value < 0.05) are plotted. Red dots indicate positive enrichment in Cyan module and blue negative; the size of the dot is inversely correlated with the FDR *q*-value and the position indicates the normalized enrichment score (NES). (a)PT, (adaptive) proximal tubule; (a)TAL, (adaptive) thick ascending limb; aEPI, adaptive epithelial; aSTR, adaptive stromal; DEGEN, degenerative.

To assess concurrent transcriptional changes in the kidney, we performed WGCNA on the microarray data generated from paired kidney samples taken during the trial (Figure 1a). Fifty-six samples were analyzed, yielding 13 modules (Figure 4a).^45^^,^^46^

A number of modules were preferentially expressed in kidney rather than in blood (Supplementary Figure S4A). These modules enriched for metabolic pathway gene sets and tubular epithelial cell signatures (Figure 4b, Supplementary Figure S4B), consistent with the abundance of tubular cells within the kidney and their absence in blood. The Darkgreen, Cyan, and Darkorange eigengene modules were downregulated with long-term CLZ treatment and upregulated in PBO-treated patients in phase A (Figure 4a). Of these 3 modules, the Cyan module was the only one that positively correlated with a higher MMDx score for AMR in the kidney (*r* = 0.31, *P* = 0.02). To assess the cell types that might be enriched in these modules, we performed cellular deconvolution, using reference gene sets obtained from a recent single cell atlas of samples taken from kidneys with acute kidney injury and chronic kidney disease.^47^ The *Cyan* module enriched for signatures defined as “adaptive tubular” and “degenerative tubular” in this study^47^ (Figure 4c), where leading edge genes include injury markers such as *MET*^48^^,^^49^, *CLU*,^50^ and *DCDC2*^51^ (Supplementary Table S8). Interpatient variability was also observed with long-term CLZ treatment (Supplementary Figure S4C). Furthermore, there was positive enrichment for an epithelial progenitor cell signature^45^ (Figure 4b), where leading edge genes include those important in epithelial regeneration (*PROM1*^52^) and epithelial cell-cell adhesion^53^ (*CLDN3*, *CLDN7*, *PAX8*, Supplementary Table S9). Of note, donor-derived cell-free DNA did not differ between groups over time (Supplementary Figure S4D), nor did it correlate with any treatment-associated modules (Figure 4a). The Purple module, which positively correlated with donor-derived cell-free DNA, contained ribosomal hub genes (Supplementary Table S10).

### CLZ-Treatment Associated With Preservation of Podocyte Signatures in AMR but Does not Affect a Kidney Module Enriched for Plasma Cell Transcripts

**Figure 5:**
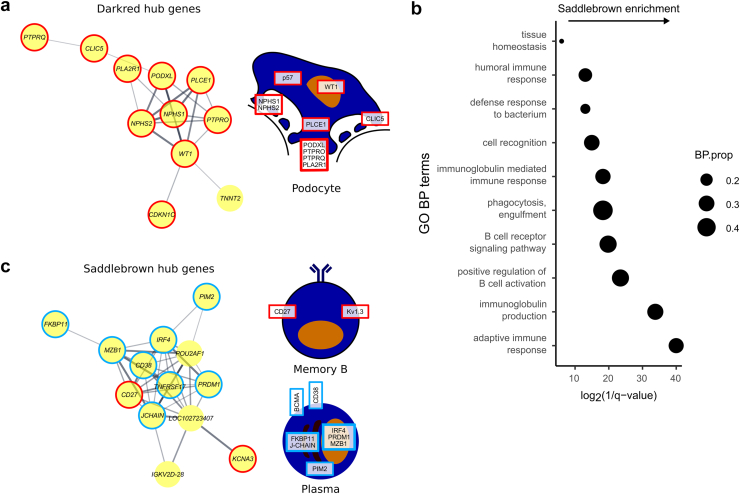
Weighted gene coexpression network analysis (WGCNA) of kidney biopsy samples. Kidney biopsies were taken alongside peripheral blood samples at week 0, week 11, and week 51 in a clazakizumab in late AMR study and analyzed by microarray. (a) STRING analysis of hub genes of the Darkred module identified with WGCNA. Hub genes were defined as those with module membership > 0.8. Only connected nodes are displayed. Edges represent confidence score > 0.4 between genes; thicker line = stronger connection. Podocyte schematic shows the location of podocyte-specific proteins encoded by the indicated genes. (b) Overrepresentation analysis of Gene Ontology (GO) biological process (BP) terms in the Saddlebrown hub genes. Ten most significant pathways are shown. Size of the dot indicates the proportion of pathway genes found within the Saddlebrown hub genes, and the position indicates the FDR *q*-value. (c) STRING analysis of hub genes of the Saddlebrown module identified with WGCNA. Hub genes were defined as those with module membership > 0.8. Only connected nodes are displayed. Edges represent confidence score > 0.4 between genes; thicker line = stronger connection. Memory B and plasma cell schematics show the location of memory B cell–specific (red) and plasma cell–specific (light blue) proteins encoded by the indicated genes.

The Darkred module, which was downregulated with PBO in phase A, enriched for podocyte and glomerular endothelial signatures (Figure 4b), containing hub genes such as *NPHS1* (encoding nephrin), *NPHS2 (*encoding podocin)*, PLA2R1*, and *WT1*, a key podocyte transcription factor (Figure 5a, Supplementary Table S11). This is consistent with damage or loss of glomeruli with ongoing AMR, and in contrast, this downregulation of Darkred module genes was not observed in patients receiving long-term CLZ treatment, suggesting glomerular preservation (Figure 4a). Interestingly, the *Saddlebrown* module enriched for humoral immunity-associated genes, including *CD38* (a plasma cell marker), *PRDM1* (encoding BLIMP1, a plasma cell-defining transcription factor), *IRF4* (a transcription factor critical for plasma cell differentiation^54^), *POU2AF1* (an IRF4-dependent transcription factor that supports GC development), *MZB1* (an effector of BLIMP1^55^), *FKBP11* (*a* plasma cell-specific antibody folding catalyst^56^), *PIM2* (a kinase required for plasmablast differentiation into plasma cells^57^), *CD27* (a memory B cell marker), and *KCNA3* (encoding the potassium channel KV1.3, which is upregulated in memory B cells^58^) (Figure 4b, 5b and c, Supplementary Table S12). This showed a positive correlation with DSA levels (Figure 4a), raising the possibility that kidney-resident plasma cells and B cells may contribute to DSA production. This module was not downregulated by long-term CLZ treatment, highlighting a potential cause of treatment failure. A summary of signatures enriched in modules correlated with treatment group can be found in Supplementary Table S13.

## Discussion

Donor-specific human leucocyte antigen antibodies promote inflammation and drive tissue injury through direct binding to endothelial cells and, via Fc-mediated effector mechanisms, activation of the classical complement pathway and of FcγR-expressing immune cells, such as monocytes, macrophages, and NK cells within the graft.^44^^,^^59^^,^^60^ Interestingly, we found that CLZ treatment reduced the expression of immune pathways related to Fc-mediated effector functions of antibody, including “Fc gamma receptor–mediated phagocytosis” and “NK cell–mediated cytotoxicity” within peripheral blood. Although a significant reduction in total circulating IgG was observed in CLZ-treated subjects in this trial,^14^ this would not account for the effects observed on FcγR-mediated pathways, since circulating monomeric IgG cannot bind these low/medium affinity Fc receptors. Rather, these FcγRs bind IgG immune complexes or deposited IgG, raising the possibility that immune cells activated within the graft vasculature via endothelial-bound DSA may recirculate in blood. In support of this conclusion, WGCNA identified a peripheral blood gene module (brown) that positively correlated with MMDx AMR score in the kidney. This module was not significantly downregulated by CLZ treatment, and enriched for monocyte signature genes, as well as “Fc gamma receptor–mediated phagocytosis” and “leucocyte transendothelial migration” gene sets. This suggests that monocytes activated within the kidney may recirculate, and can be detected in blood. Of note, microvascular inflammation is a histological hallmark of AMR, where neutrophils and mononuclear cells accumulate within the lumen of peritubular capillaries.^4^^,^^61^ The fate of these cells is unknown, and challenging to track directly in humans; however, our data raise the possibility that at least some of these cells do not die *in situ*. This would explain why and how transcriptional changes in peripheral blood may have utility in monitoring AMR-associated processes in the kidney.

Gene sets significantly downregulated in peripheral blood by CLZ treatment included B-cell activation and JAK-STAT signaling pathway genes, as well as gene modules that enriched for monocyte, platelet, erythrocyte, and Tfh signatures. Tfh are critical for the progression of germinal center responses and the generation of mutated, high-affinity antibodies, including in AMR, where they produce IL-21 upon stimulation with donor antigen and can activate B cells.^62^ IL-6 is required for the differentiation of CD4 T cells into polarized Tfh,^63^ and our findings suggest that effects of CLZ on Tfh may contribute to the reduction in DSA observed in the phase 2 study.^14^ Although the total platelet number did not differ between CLZ and PBO groups in the clinical trial,^14^ the observed downregulation of platelet-associated genes is consistent with known effects of IL-6 on platelet activation,^40^^,^^41^ and points to a previously unappreciated aspect of CLZ efficacy in AMR, because platelets augment early inflammation in response to DSAs in animal models of AMR.^15^

In the kidney, we found that a module enriched with damaged tubular and progenitor signatures (presumably reflecting tubular injury and attempts at repair following antibody-mediated injury), was downregulated by long-term CLZ. In concert, a podocyte gene–rich module was preserved with CLZ but downregulated with PBO, suggesting relative preservation of glomeruli with CLZ. A recent single-cell RNA-sequencing study emphasized transcriptional changes occurring in kidney tubular and structural cells during rejection.^64^

Remarkably, the Saddlebrown kidney module positively correlated with serum DSA levels and strongly enriched for plasma cell–associated genes, raising the possibility that kidney-resident plasma cells may contribute to circulating DSA. Consistent with this, a previous study observed that the presence of plasma cells in the graft was significantly associated with positive C4d staining and anti–human leucocyte antigen antibodies in serum and graft eluates.^65^ Tertiary lymphoid aggregates that support alloantibody generation have been described within kidney allografts in humans and mouse,^66^ and may represent a source of alloantibody that is not effectively targeted by CLZ. However, the Saddlebrown module was negatively correlated in PBO-treated patients, highlighting the complexity and heterogeneity of the disease and responses to treatment in different individuals.

Finally, our study highlights 2 potential contributors to the early termination of the IMAGINE trial^17^^,^^18^ (https://clinicaltrials.gov/study/NCT03744910) in addition to significantly reduced CLZ dosing. First, CLZ treatment failed to significantly reduce the plasma cell gene rich kidney module, suggesting that either the antibody does not access these tissue niches well, or that the plasma cells in the kidney rely on other survival factors such as BAFF or APRIL. Second, we found substantial interpatient variability in the extent to which CLZ led to downregulation of antibody effector gene sets (Fc gamma receptor–mediated phagocytosis, NK cell–mediated cytotoxicity) in peripheral blood in the longer term, with some patients even showing a rebound increase in these gene sets after 12 months of treatment, despite showing a reduction in DSA. This variability could reflect incomplete treatment adherence or development of antidrug antibodies but may also reflect our increasing understanding that AMR is a heterogeneous condition. This suggests that, for a subset of patients, additional treatments may be required that target antibody-effector function rather than DSA generation; and emphasize that our current “one size fits all” approach to treatment of AMR has inevitable limitations. Our results highlight why in-depth molecular assessment of patients involved in clinical trials has value in informing future efforts to find effective therapeutic strategies for this difficult condition.

Overall, our results provide potential mechanistic insights into the effects, and limitations, of IL-6 neutralization in humans in the context of AMR. Beyond this, we find that transcriptional changes in peripheral blood may provide a readout of tissue antibody-mediated FcγRs ligation, suggesting that the assessment of peripheral blood may have potential utility in monitoring AMR-associated processes and therapeutic efficacy in the kidney. However, further mechanistic research in larger sample sizes will be necessary to draw robust conclusions.

## Disclosure

RZ is an NIHR Clinical Academic Fellow. CYCL was funded by the Gates Cambridge scholarship trust and University of Cambridge School of Clinical Medicine Elmore fund. MRC was supported by an National Institute of Health Research (NIHR) Research Professorship (RP-2017-08-ST2-002); a Wellcome Trust Investigator Award (220268/Z/20/Z); the NIHR Blood and Transplant Research Unit in Organ Donation (NIHR203332), a partnership between NHS Blood and Transplant, University of Cambridge, and Newcastle University; the NIHR Cambridge Biomedical Research Centre (NIHR203312); and a Wellcome Discovery Award (227890/Z/23/Z). GAB reports grants from HI-Bio/Biogen, USA, where he is an advisor. He is also an advisor to Argenx, Belgium. KB reports grants from CSL Behring and personal fees from CSL Behring during the conduct of the study; grants from Alexion, Astellas, AstraZeneca, Chiesi, CSL Behring, MSD, Otsuka, Stada, Takeda. PH reports grants from Natera, Inc. and has an interest/has advised in One Lambda Inc., Transcriptome Sciences Inc., Argenx BV and Alexion Pharmaceuticals Inc. All other authors declare no conflicting interests.

## Supplementary Material

Supplementary File (PDF and .xlsx) **Supplementary Methods**. Gene sets used for enrichment analysis. Module preservation statistics for WGCNA modules. **Supplementary References. Figure S1.** Effects of clazakizumab on the peripheral blood transcriptome in late antibody-mediated rejection (AMR). **Figure S2.** Clinical characteristics of individuals in the clazakizumab arm who experienced rebound with long-term clazakizumab (phase B). **Figure S3.** Weighted gene coexpression network analysis (WGCNA) of peripheral blood samples. **Figure S4.** Weighted gene coexpression network analysis (WGCNA) of kidney biopsy samples. **Table S1.** Metadata for *n* = 58 peripheral blood samples taken alongside the clazakizumab in late AMR trial (separately uploaded file, “.xlsx”). **Table S2.** Leading edge genes for enrichment of “FcγR-mediated phagocytosis” in peripheral blood samples following 12 weeks of clazakizumab treatment in phase A. **Table S3.** Leading edge genes for enrichment of “natural killer (NK) cell–mediated cytotoxicity” in peripheral blood samples following 12 weeks of clazakizumab treatment in phase A. **Table S4.** Baseline AMR parameters comparing those with molecular evidence of rebound with those without. **Table S5.** Lightyellow module hub genes (module membership > 0.8) identified from WGCNA of peripheral blood mononuclear cells. **Table S6.** Brown module hub genes (module membership > 0.8) identified from WGCNA of peripheral blood mononuclear cells. **Table S7.** Gene ontology analysis of brown module hub genes identified from WGCNA of peripheral blood mononuclear cells. **Table S8.** Leading edge genes for enrichment of “adaptive proximal tubule” signature^47^ in the cyan module identified in kidney samples with WGCNA. **Table S9.** Leading edge genes for enrichment of “epithelial progenitor cell” signature^45^ in the cyan module identified in kidney samples with WGCNA. **Table S10.** Purple module hub genes (module membership > 0.9) identified from WGCNA of kidney samples. **Table S11.** Darkred module hub genes (module membership > 0.8) identified from WGCNA of kidney samples. **Table S12.** Saddlebrown module hub genes (module membership > 0.8) identified from WGCNA of kidney samples. **Table S13.** Summary of enrichments correlated with treatment phase from WGCNA analysis of blood and kidney samples.
