# Supplementary material for: The Effects of Clazakizumab on Peripheral Blood and Kidney Transcriptomes in Patients With Late Antibody-Mediated Rejection
Source: Kidney Int Rep. 2025 Aug 26;10(11):4027–40. doi: 10.1016/j.ekir.2025.08.027 (PMC12640031; doi:10.1016/j.ekir.2025.08.027)
Supplement: Supplementary File (PDF and .xlsx) — Supplementary Methods. Gene sets used for enrichment analysis. Module preservation statistics for WGCNA modules. Supplementary References. Figure S1. Effects of clazakizumab on the peripheral blood transcriptome in late antibody-mediated rejection (AMR). Figure S2. Clinical characteristics of individuals in the clazakizumab arm who experienced rebound with long-term clazakizumab (phase B). Figure S3. Weighted gene coexpression network analysis (WGCNA) of peripheral blood samples. Figure S4. Weighted gene coexpression network analysis (WGCNA) of kidney biopsy samples. Table S1. Metadata for n = 58 peripheral blood samples taken alongside the clazakizumab in late AMR trial (separately uploaded file, “.xlsx”). Table S2. Leading edge genes for enrichment of “FcγR-mediated phagocytosis” in peripheral blood samples following 12 weeks of clazakizumab treatment in phase A. Table S3. Leading edge genes for enrichment of “natural killer (NK) cell–mediated cytotoxicity” in peripheral blood samples following 12 weeks of clazakizumab treatment in phase A. Table S4. Baseline AMR parameters comparing those with molecular evidence of rebound with those without. Table S5. Lightyellow module hub genes (module membership > 0.8) identified from WGCNA of peripheral blood mononuclear cells. Table S6. Brown module hub genes (module membership > 0.8) identified from WGCNA of peripheral blood mononuclear cells. Table S7. Gene ontology analysis of brown module hub genes identified from WGCNA of peripheral blood mononuclear cells. Table S8. Leading edge genes for enrichment of “adaptive proximal tubule” signature47 in the cyan module identified in kidney samples with WGCNA. Table S9. Leading edge genes for enrichment of “epithelial progenitor cell” signature45 in the cyan module identified in kidney samples with WGCNA. Table S10. Purple module hub genes (module membership > 0.9) identified from WGCNA of kidney samples. Table S11. Darkred module hub genes (module membership > 0.8) identified f [file mmc1.pdf]

Supplementary Material: The effects of clazakizumab on peripheral blood and kidney transcriptomes in patients with late antibody-mediated rejection

Authors: Roy Zhang[1,2], Colin Y.C. Lee[1,2,3], Martina Schatzl[4], Klemens Budde[5], Fabian Halleck[5], Bernd Jilma[6], Jessica Chang[7], Philip Halloran[7], Georg A. Böhmig[4], Menna R. Clatworthy[1,2,3]*

Affiliations:

[1]Molecular Immunity Unit, University of Cambridge Department of Medicine, Cambridge, UK.

[2]Cambridge Institute for Therapeutic Immunology and Infectious Diseases

[3]Cellular Genetics, Wellcome Sanger Institute, Hinxton, UK.

[4]Division of Nephrology and Dialysis, Department of Medicine III, Medical University of Vienna, Vienna, Austria

[5]Department of Nephrology, Charité Universitätsmedizin Berlin, Berlin

[6]Department of Clinical Pharmacology, Medical University of Vienna, Vienna, Austria

[7]Alberta Transplant Applied Genomics Centre, University of Alberta, Edmonton, Alberta, Canada.

*Corresponding author.

Address for correspondence:

Molecular Immunity Unit

University of Cambridge Department of Medicine

MRC Laboratory of Molecular Biology,

Cambridge Biomedical Campus,

Francis Crick Avenue,

Cambridge. CB2 0QH.

Phone: 44-1223-267279

Email: mrc38@cam.ac.uk

Supplementary Methods

Gene sets used for enrichment analysis

Hallmarks signatures (v2023.2) and Kegg signatures (kegg_legacy.v2023.2) were obtained from MSigDB[S1,S2]. The immune complex-stimulated macrophage signature was derived from murine bone marrow-derived macrophages[S3]. For cell-type enrichment, peripheral blood mononuclear cell gene sets[S4] and kidney-specific gene sets[S5] were derived from single-cell sequencing analysis. T follicular helper cell signatures were derived from Weinstein *et al*.[S6]. Neutrophil signatures were derived from Xie *et al*.[S7]. Adaptive and degenerative tubular signatures were derived from Lake *et al*.[S8]. Plasma cell signatures were derived from Zhang *et al*.[S9].

Module preservation statistics for WGCNA modules

Module preservation analysis was performed[S10], with multiple preservation statistics assessing module density and intramodular connectivity calculated and significance evaluated using a permutation test to generate *Z*-scores (200 permutations). These were aggregated into a summary statistic $Z_{summary}$, where >10 indicates strong evidence for preservation and <2 indicates weak evidence for preservation. Since $Z_{summary}$ tends to increase with module size, the composite *medianRank* statistic is also provided where a lower rank indicates greater relative preservation. Modules with over 1000 genes were reduced by randomly sampling 1000 genes.

Supplementary Tables

***Supplementary Table S1*: Metadata for n=58 peripheral blood samples taken alongside the clazakizumab in late AMR trial (separately uploaded file).**

Definitions where required:

sample	Sample ID
individual	Patient ID
treatment arm	Whether patient received placebo or clazakizumab during Phase A of trial
date of index sample	Date when week 0 sample was taken
donor type	Whether kidney donor was a living or deceased donor organ
donor age	Age of donor at the point of retrieval
immunosuppression	Immunosuppression regime at recruitment to trial<br>Cyclospoine A, CyA; mycophenolate mofetil, MMF; tacrolimus, Tac; prednisolone, Pred; methylprednisolone, Methylpred; enteric-coated mycophenolic acid, EC-MPA.
AMR MMDx	Score for antibody-mediated rejection by the molecular microscope diagnostic system[S11,S12].
AMR BANFF	1 – 'Chronic active ABMR' as per BANFF 2017 update[S13].<br>0 – 'Chronic ABMR' as per BANFF 2017 update, defined as transplant glomerulopathy without evidence of current/recent antibody interaction with the endothelium, but with a prior documented diagnosis of chronic active ABMR.
EGFR	Estimated glomerular filtration rate
DSA	Donor-specific antibody levels, as a percentage of levels at week 0.
IgG	Immunoglobulin G levels, as a percentage of levels at week 0.
batch	Sequencing batch

***Supplementary Table S2*: Leading edge genes for enrichment of '*FcγR-mediated phagocytosis*' in peripheral blood samples following 12 weeks of clazakizumab treatment in Phase A.**

PIK3CA	PAK1	AKT2	AKT1	DNM1L	LIMK2
ASAP2	PTPRC	ARPC1B	WASF2	WASF1	MAPK3
DNM3	PIP5K1B	SYK	RAF1	ARPC2	PLPP3
ASAP1	GSN	VAV1	PIK3CB	PIK3R1	INPP5D
CRK	CRKL	GAB2	FCGR3A	MARCKS	MARCKSL1
MAPK1	ARPC5	FCGR2A	NCF1	ARPC1A	ARF6
VASP	DOCK2	PIKFYVE	PRKCB	ARPC3	DNM1
LYN	ARPC4	ASAP3	PIK3CG	RPS6KB2	WAS
WASF3	HCK	PIK3R2	PIP4K2B	PRKCD	PIK3CD
CDC42	RAC1	PLCG2	RPS6KB1	FCGR1A	

***Supplementary Table S3*: Leading edge genes for enrichment of '*natural killer (NK) cell-mediated cytotoxicity*' in peripheral blood samples following 12 weeks of clazakizumab treatment in Phase A.**

PIK3CA	FAS	RAC1	PIK3R2	PLCG2	PRKCB
FCGR3B	GRB2	BRAF	ITGB2	IFNGR2	PIK3CG
MAPK1	PPP3R1	SYK	PPP3CA	RAF1	SOS2
KRAS	NFATC3	VAV1	PPP3CB	PIK3CB	CHP1
PAK1	PTPN11	MAP2K2	IFNAR1	FCGR3A	BID
TNFRSF10C	NRAS				

***Supplementary Table S4*: Baseline AMR parameters comparing those with molecular evidence of rebound with those without.**

Parameter at baseline	No rebound (n=6)	Rebound (n=3)	*P*-value
**Serological assessment**			
MFI, mean (SD)	11833 (7669)	5494 (4274)	0.37
**Morphological AMR lesions and scores**			
Glomerulitis (g score ≥ 1), n (%)	4 (0.67)	3 (1)	
g score, median	2	2	0.89
Peritubular capillaritis (ptc score ≥ 1), n (%)	4 (0.67)	2 (0.67)	
ptc score, median	1.5	2	0.89
Transplant glomerulopathy (cg score ≥ 1), n (%)	4 (0.67)	3 (1)	
cg score, median	2.5	2	1.00
C4d in peritubular capillaries (C4d score ≥ 1), n (%)	4 (0.67)	0 (0)	
C4d score, median	1.5	0	0.12
Interstitial fibrosis (ci score ≥ 1), n (%)	5 (0.83)	2 (0.67)	
ci score, median	2	1	0.35
Tubular atrophy (ct score ≥ 1), n (%)	5 (0.83)	2 (0.67)	
ct score, median	1.5	1	0.28
Vascular fibrosis intimal thickening (cv score ≥ 1), n (%)	3 (0.75)	2 (0.67)	
cv score, median*	1.5	1	0.45
**Molecular classifiers of rejection and injury**			
AMR score, median	0.69	0.81	0.37
“All rejection” score, median	0.73	0.67	0.90

AMR, antibody-mediated rejection; MFI: mean fluorescence intensity; DSA, donor-specific antibody. *P*-values calculated using Wilcoxon rank sum test.

*cv score was not available for 2 recipients who did not rebound.

***Supplementary Table S5*: Lightyellow module hub genes (module membership > 0.8) identified from WGCNA of peripheral blood mononuclear cells.** Module membership is the correlation between gene expression profile and module eigengene.

ITGB5	TUBB1	PARD3	GP1BB	DNM3	CALD1
PDE5A	GP9	GNAZ	SYTL4	TPM4	PKHD1L1
SELP	RHOBTB1	PPBP	RAB27B	SEPTIN5	DAB2
CTTN	CAVIN2	MYLK	PTCRA	ARHGAP18	VEPH1
GUCY1B1	PF4	ANO6	MMD	TREML1	PDGFA
ITGB3	PCSK6	LINC00989	BEND2	LY6G6F	PROS1
MFAP3L	PDE3A	RUFY1	PLOD2	CMTM5	GP6
CTDSPL	F13A1	P2RY12	CFAP161	PRUNE1	MPST
GNG11	PRKAR2B	PGRMC1	ITGA2B	LIMS1	CABP5
SH3BGRL2	MMRN1	SPARC	LGALSL	ARHGAP6	LIPH
ELOVL7	PTGS1	TRAPPC3L	ABLIM3	SH3TC2	ESAM
LTBP1	C2orf88	ABCC3	STON2	CLU	FSTL1
EGF					

***Supplementary Table S6*: Brown module hub genes (module membership > 0.8) identified from WGCNA of peripheral blood mononuclear cells.** Module membership is the correlation between gene expression profile and module eigengene.

RNF149	CDC123	LAMTOR3	MAPK3	MCTP1	PPP2CA
ZFYVE16	SLC2A3	MXD1	TMBIM4	FGD4	SYK
FBXL5	GK	GBE1	PLAUR	S100A8	CFLAR
RP2	S100A9	CKLF	ITPRID2	MIER1	IL6R
RNF130	FRAT1	PIP4P2	STAG2	ANTXR2	SERINC1
ACAP2	IFRD1	KIF13A	RB1CC1	MAPK14	B9D2
CMTM6	H3F3B	ST6GALNAC2	SNX18	PELI1	VNN3
EXOC6	LBR	TMEM154	ELF1	RRAGD	NFIL3
GCA	MCTP2	KCNE3	LRG1	RHOA	CPQ
ACTR3	CD63	BST1	ZNF148	PSMB3	IFITM2
NAMPT	ACSL4	TMEM167B	DENND10	CCDC71L	PPP1R15B
CAB39	ARPC3	ROCK1	TMEM71	RBP7	ACOX1
FAM49B	LPCAT2	IKBIP	ATP6V1A	CASC4	TMEM33
GMPR2	RALB	HIF1A	CD53	WTAP	MBD4
LAMP2	GNAI3	CAPZA2	HECW2	NIBAN1	FBXO38
CCPG1	NUP58	ELOVL5	PPP4R1	IFNAR1	YTHDF3
STXBP3	HCK	MAP2K4	PACSIN2	SPAG9	SIPA1L2
PRKAR1A	PLXNC1	ST8SIA4	RAB1A	MYL12B	CD58
CAPZA1	OSTF1	PPM1B	MAP3K5	ATF6	LPGAT1
QPCT	RNF146	AFTPH	SDCBP	CTSS	PLBD1
WASHC4	ARPC5	RBPJ	CD46	AC015871.1	
SPOPL	HBP1	TGFBR1	APC	SHOC2	VAMP3

AQP9	CACUL1	PLIN3	PPP1R12A	TPD52L2	NR3C1
RAB18	TOR1AIP1	NPTN	RSBN1L	JAML	FAM49A
PJA2	UBE2W	PRCP	ELF2	STAM2	CMTM2
STK38L	BCL2A1	VNN2	ALOX5AP	ZBTB34	XRN2
MNDA	SQOR	UBXN2B	ASAH1	ATP6V0E1	VAV1
CYB5R4	IL10RB	IGSF6	RGS2	KIAA0040	LMNB1
MCL1	LCP1	TDP2	TPM3	RASSF2	GLIPR1
RAB8B	BASP1	ATP6V1C1	ABI1	RAB33B	TGFA
TLR1	LRRK2	PTTG1IP	VAPA	TSEN34	UBE2D1
RIT1	CXCR1	TMEM30A	CEBPD	DIP2B	KBTBD2
JPT1	MSL1	SOD2	FPR1	ALOX5	MSRB1
PPP1R3B	OGFRL1	ABHD5	RAB5IF	NT5C2	DOCK11
SRGN	B4GALT5	ACTR2	STK17B	C5AR2	HMGB2
TLR8	BAZ1A	HSD17B11	PTPRC	GALNT3	CRLF3
CTBS	QKI	SNX13	ANXA3	ACSS3	SLK
ARPC2	GMFG	SNX10	AGO4	FAM126B	RESF1
TLR4	F2RL1	BACH1	STEAP4	ATP6V0D1	ROPN1L
SP3	PHF20L1	CD55	GNAQ	ZMPSTE24	MPZL3
ETS2	LAT2	PELI2	PGK1	EGLN1	ACSL1
MOSPD2	RRM2B	RNASEL	RTF2	ZNF281	FPR2
KLHL2	BNIP2	VPS4B	H3F3A	PYGL	ATP6V1B2
TM6SF1	DHRS7	SNAP23	IPMK	KCNJ2	MCMBP
ANP32A	CHUK	CEP19	CLIC1	GNA13	OSBPL8
PSEN1	GRB2	RAB2A	FAR1	DNAJC3	SPAST
ITM2B	LIN7A	RAB27A	UHRF1BP1L	ARID4A	PGD
NCF2	CNEP1R1	FCGR3B	OSBPL2	TMCC3	SERPINB1
CRK	PPP4C	CASP4	ZNF267	RNF13	FRAT2
FCGR2A	BOD1L1	IFNGR2	DGAT2	IFNGR1	KIF5B
DCP2	PGM2	MFSD14A	HSDL2	LRMP	DAPP1
MAP3K2	KIAA0232	SMCHD1	KCNJ15	COP1	RAB31
GLIPR2	PTEN	RCBTB2	GPAT3	HNRNPH2	MARCH7

***Supplementary Table S7*: Gene ontology analysis of brown module hub genes identified from WGCNA of peripheral blood mononuclear cells.** Biological process terms defined by Gene Ontology are included. Anno and Sig indicate annotated and significant genes respectively. Fisher.elim indicates *P*-values derived from Fisher's exact test and using the elim algorithm, designed to be more conservative[S14].

BP.GO.ID	BP.Term	Anno	Sig	Fisher.elim
GO:0019432	triglyceride biosynthetic process	10	7	0.00027
GO:1901568	fatty acid derivative metabolic process	10	7	0.00027
GO:0001774	microglial cell activation	10	7	0.00027
GO:0006801	superoxide metabolic process	13	8	0.00034
GO:0030593	neutrophil chemotaxis	19	10	0.00034
GO:0071346	cellular response to interferon-gamma	19	10	0.00034
GO:0048708	astrocyte differentiation	11	7	0.00063
GO:0006631	fatty acid metabolic process	31	13	0.00075
GO:0016050	vesicle organization	49	17	0.00159
GO:0010256	endomembrane system organization	79	24	0.00159
GO:0009617	response to bacterium	75	23	0.00173
GO:2000379	positive regulation of reactive oxygen s...	19	9	0.00182
GO:0061640	cytoskeleton-dependent cytokinesis	16	8	0.00214
GO:0032757	positive regulation of interleukin-8 pro...	13	7	0.00241
GO:0045010	actin nucleation	10	6	0.00246
GO:0031341	regulation of cell killing	10	6	0.00246
GO:0010033	response to organic substance	388	89	0.00264
GO:0071674	mononuclear cell migration	24	10	0.0033
GO:0140029	exocytic process	17	8	0.00347
GO:0014855	striated muscle cell proliferation	11	6	0.00465
GO:0060419	heart growth	18	8	0.00535
GO:0001837	epithelial to mesenchymal transition	18	8	0.00535
GO:0045165	cell fate commitment	22	9	0.00612
GO:0051640	organelle localization	87	24	0.0065
GO:0046330	positive regulation of JNK cascade	15	7	0.00665
GO:0023061	signal release	51	16	0.0068
GO:0009628	response to abiotic stimulus	126	32	0.00706
GO:0098771	inorganic ion homeostasis	83	23	0.00723
GO:0044283	small molecule biosynthetic process	43	14	0.00779
GO:0043299	leukocyte degranulation	19	8	0.00792
GO:0070665	positive regulation of leukocyte prolife...	19	8	0.00792
GO:1903426	regulation of reactive oxygen species bi...	12	6	0.008
GO:0042060	wound healing	79	22	0.00803
GO:0051347	positive regulation of transferase activ...	79	22	0.00803
GO:0007166	cell surface receptor signaling pathway	325	70	0.00808
GO:0006954	inflammatory response	103	27	0.0084

GO:0070374	positive regulation of ERK1 and ERK2 cas...	27	10	0.00886
GO:0070887	cellular response to chemical stimulus	398	93	0.00893
GO:0045937	positive regulation of phosphate metabol...	128	32	0.00906
GO:0031349	positive regulation of defense response	44	14	0.00973
GO:0030003	cellular cation homeostasis	71	20	0.0099
GO:0002532	production of molecular mediator involve...	16	7	0.01015
GO:0002688	regulation of leukocyte chemotaxis	16	7	0.01015
GO:0032946	positive regulation of mononuclear cell ...	16	7	0.01015
GO:0050671	positive regulation of lymphocyte prolif...	16	7	0.01015
GO:0006935	chemotaxis	69	25	0.01088
GO:0071902	positive regulation of protein serine/th...	32	11	0.01148
GO:0042742	defense response to bacterium	32	11	0.01148
GO:0030030	cell projection organization	166	39	0.0123
GO:0002443	leukocyte mediated immunity	54	16	0.01234

***Supplementary Table S8*: Leading edge genes for enrichment of '*adaptive proximal tubule*' signature[S8] in the cyan module identified in kidney samples with WGCNA.**

AIF1L	GNAS	HMGB1	ANP32B	NFE2L2	HLA-E
MAL	H3F3A	SCAF11	SPINT1	RPS21	HLA-B
CKB	WFDC2	C19orf33	TMEM123	HLA-A	RPL10
COMT	PTTG1IP	EIF1	VPS25	LBR	CRIP2
MAL2	KRT19	RNPS1	PHF3	RPS3	GSTP1
AKAP9	CD46	RPS27A	GCC2	MYL6	DYNLL1
CLDN7	BCLAF1	N4BP2L2	RPS15A	STMN1	RPS2
CD24	TMBIM4	CLU	MET	RPS9	TAPBP
JUP	KRT7	CD81	HNRNPH1	RPL35	ITGB1
SYNE2	NR2F2	ZRANB2	PLSCR1	S100A16	TUBA1A
CPVL	TSC22D1	SPCS2	BCL7C	CLIC1	PIGR
DYNC2LI1	DDX17	LRP10	NKTR	SRRM2	RPS8
DSG2	SRI	GOLGB1	RCN2	RPL34	RPL37
CD9	GPBP1	CHD9	H1FX	CAPG	ORMDL1
LAPTM4B	KTN1	LUC7L3	RPL11	RPL8	RPSA
PTPRF	BICC1	GPRC5C	NTN4	RPL30	MDM4
FLNB	HSBP1	RPS6	TPR	FZD1	FAU
DDR1	YWHAZ	JUN	IFITM2	MDK	MMP7
REEP5	SUMO2	RPS3A	SF1	ZFAS1	IFITM3
SPINT2	CLDN3	PRRC2C	RPL35A	PNISR	ERP29
EPCAM	GTF2I	ATRX	PRMT1	TPM4	DSP
DMKN	HMGN1	ENO1	TNFSF10	ADAM10	RPL12
TSPAN6	LAMB1	RPL41	PARP14	ANXA2	TMSB4X
PKHD1	ATP6AP2	PLXNB2	CAST	SEC11A	MYH9
MRPL33	HNRNPDL	RPS23	MYL12A	COL27A1	KLF6
HNRNPU	ATRAID	PKN2	RPL10A	LSM7	UBC
MACC1	MGAT4B	S100A13	KMT2E	LTBP3	WASF2
CD59	SON	APP	RPL24	SSR2	RBM25
PCM1	IER3	PNN	PSMB8	FAT1	SERPING1
WSB1	BCAM	DCDC2	MYL12B	RPS12	RPL28
ANXA11	B2M	BTF3	EIF5B	HSP90B1	XRCC5
RASSF7	HNRNPA1	TNFRSF11B	IFI27L2	TMEM50A	EIF3G
FUS	JAK1	NACA	RPL23A	RPS27	PSME1
AGRN	RPL5	NPM1	RPS4X	ACTB	LEPROT
PAX8	RPS24	PKM	EIF3A	BPTF	NFKBIA
ARHGAP29	HNRNPA2B1	AHNAK	RPL7A	ACTG1	TMEM179B
ITM2C	SET	S100A10	ITGB8	CALR	RPL32
OXR1	RAC1	RBMX	ARF5	BANF1	TPM1
RBM39	ARGLU1	SNRPE	EIF3D	RPL19	MARCKSL1
SYNGR2	KRT10	SOX4	PRPF4B	TMEM205	ZBTB20
PLXNB1	IQGAP1	DHRS7	RPL6	RPLP1	RPL3
PROM1	HLA-C	FOS	TSPO	TUSC3	SSR4
LRRFIP1	SRSF7	RPL21	IER2	TUBA1B	PPP1R14B
S100A6	RSRC2	YWHAH	MALAT1	S100A11	ANXA5

PON2	HSPA5	C9orf3	YBX3	RPL18	LSM2
RPL15	ZFP36L1	RPS14	KMT2A	PDIA6	IFI27
CLDN4	IGFBP7				

***Supplementary Table S9*: Leading edge genes for enrichment of '*epithelial progenitor cell*' signature[S5] in the cyan module identified in kidney samples with WGCNA.**

SLC5A3	POU3F3	TACSTD2	PERP	SLPI	WNT10A
IRX2	CLDN7	CPVL	PKHD1	ITM2C	KRT7
TSPAN8	DEGS2	PLEKHH2	PAX8	KIF12	CHL1
PFKFB3	CLDN10	SPINT2	CLDN19	PROM1	SOD3
CYS1	LIX1	EPCAM	LMO7	WFDC2	CLDN3
S100A14					

***Supplementary Table S10*: Purple module hub genes (module membership > 0.9) identified from WGCNA of kidney samples.** Module membership is the correlation between gene expression profile and module eigengene.

RPL35	RPL11	RPL30	PFDN5	RPL24	RPL23A
C4orf46	RPS11	FAU	RPL18A	RPL23	RPS16
RPL8	RPL32	NXT1	RPL18	RPL31	RPL34
TMEM258					

***Supplementary Table S11*: Darkred module hub genes (module membership > 0.8) identified from WGCNA of kidney samples.** Module membership is the correlation between gene expression profile and module eigengene.

CLIC5	HTRA1	SPOCK1	TENM2	ALS2CL	DPP6
NPHS1	NTNG1	WT1	KLK7	NPHS2	TYRO3
PLA2R1	GJA3	TSPAN2	PODXL	SNCA	CHRDL1
PLCE1	FMN2	CDKN1C	TNNT2	F3	NRIP2
PTPRO	NDNF	ADAMTS19	FGF1	PTPRQ	SPOCK2
ST6GALNAC3	PCOLCE2				

***Supplementary Table S12*: Saddlebrown module hub genes (module membership > 0.8) identified from WGCNA of kidney samples.** Module membership is the correlation between gene expression profile and module eigengene.

MZB1	AC006548.20	ANKRD36BP2	IGLJ3	IGH	IGK

TNFRSF17	IGKV3-20	IGLV1-40	IGKV2-28	IGLV3-10	IGKV1-12
POU2AF1	IGKC	PIM2	IGKV3-11	FER1L4	CD38
IGKV1-5	IRF4	IGLV1-41	IGJ	IGHM	KCNA3
IGLL3P	IGHG3	IGLV1-44	IGHG1	IGHA1	PDK1
RING1	IGKV1-39	CD27	KIAA0125	PRDM1	FKBP11

***Supplementary Table S13*: Summary of enrichments correlated with treatment phase from WGCNA analysis of blood and kidney samples.** Enrichments included in manuscript together with the modules correlated with treatment phase (in brackets). Arrows indicate positive or negative correlation with treatment phase. PBO, placebo group phase A; PBO-CLZ, placebo group phase B; CLZ, clazakizumab group phase A; CLZ-CLZ, clazakizumab group phase B; NS, non-significant**.**

<table>
<tr><th></th><th>Blood</th><th>Kidney</th></tr>
<tr><td>PBO</td><td>↓Leucocyte transendothelial migration (Brown, NS)
↓Fc gamma receptor-mediated phagocytosis (Brown, NS)</td><td>↑Adaptive and degenerative tubular (Cyan)
↑Epithelial progenitor cell (Cyan)
↓Podocyte, glomerular endothelial cell (Darkred)
↓Plasma cell (Saddlebrown)</td></tr>
<tr><td>PBO-CLZ</td><td rowspan="2">↓Platelet activation (Lightyellow)
↓T follicular helper cell (Lightyellow)
↓Leucocyte transendothelial migration (Brown, NS)
↓Fc gamma receptor-mediated phagocytosis (Brown, NS)</td><td>↓Adaptive and degenerative tubular (Cyan)
↓Epithelial progenitor cell (Cyan)
↑Plasma cell (Saddlebrown)</td></tr>
<tr><td>CLZ</td><td>↓Podocyte, glomerular endothelial cell (Darkred)</td></tr>
<tr><td>CLZ-CLZ</td><td>↑Leucocyte transendothelial migration (Brown, NS)
↑Fc gamma receptor-mediated phagocytosis (Brown, NS)</td><td>↓Adaptive and degenerative tubular (Cyan)
↓Epithelial progenitor cell (Cyan)</td></tr>
</table>

Legends to Supplementary Figures

***Supplementary Figure S1*: Effects of clazakizumab on the peripheral blood transcriptome in late antibody-mediated rejection (AMR)**. Peripheral blood samples were taken at week 0, week 12 and week 52 in a clazakizumab in late AMR study and analyzed by RNA sequencing (RNA-seq). Abbreviations: NES, normalized enrichment score; GSEA, gene set enrichment analysis; CLZ, clazakizumab; PBO, placebo.

a) GSEA of the differential expression analysis of week 12 versus week 0 samples in the placebo arm, using the hallmarks pathways (MSigDB). Only significant pathways (FDR *q*-value < 0.05) are plotted. Red dots indicate pathways upregulated during Phase A in the placebo arm; blue indicates downregulated pathways. The size of the dot is inversely correlated with the FDR *q*-value and the position indicates the normalized enrichment score (NES).

b) GSEA of the differential expression analysis of week 12 versus week 0 samples in the clazakizumab arm, using the hallmarks pathways (MSigDB). Only significant pathways (FDR *q*-value < 0.05) are plotted. Red dots indicate pathways upregulated during Phase A in the clazakizumab arm; blue indicates downregulated pathways. The size of the dot is inversely correlated with the FDR *q*-value and the position indicates the normalized enrichment score (NES).

c) GSEA of the differential expression analysis of week 52 versus week 12 samples in the placebo arm, using the hallmarks pathways (MSigDB). Only significant pathways (FDR *q*-value < 0.05) are plotted. Red dots indicate pathways upregulated during Phase B in the placebo arm; blue indicates downregulated pathways. The size of the dot is inversely correlated with the FDR *q*-value and the position indicates the normalized enrichment score (NES).

d) GSEA of the differential expression analysis of week 52 versus week 12 samples in the clazakizumab arm, using the hallmarks pathways (MSigDB). Only significant pathways (FDR

q-value < 0.05) are plotted. Red dots indicate pathways upregulated during Phase B in the clazakizumab arm; blue indicates downregulated pathways. The size of the dot is inversely correlated with the FDR *q*-value and the position indicates the normalized enrichment score (NES).

***Supplementary Figure S2*: Clinical characteristics and molecular changes in the kidney of individuals in the clazakizumab arm who experienced rebound in peripheral blood transcriptomic signatures with long-term clazakizumab (phase B)**.

a) Scatterplot indicating donor-specific antibody (DSA) levels taken at 3 time points in the clazakizumab in late antibody-mediated rejection study. DSA levels are plotted as a percentage of baseline (week 0). Lines connect samples belonging to individuals. Blue indicates samples belonging to 3 individuals that experienced rebound following long-term clazakizumab use in Phase B; red indicates samples belonging to those who did not experience rebound.

b) Scatterplot indicating estimated glomerular filtration rate (eGFR) levels taken at 3 time points in the clazakizumab in late antibody-mediated rejection study. Lines connect samples belonging to individuals. Blue indicates samples belonging to 3 individuals that experienced rebound following long-term clazakizumab use in Phase B; red indicates samples belonging to those who did not experience rebound.

c) GSEA of the differential expression analysis of week 52 kidney samples, comparing individuals who experienced rebound in peripheral blood transcriptomic signatures versus those who did not, using the KEGG pathways. Red dots indicate pathways upregulated during phase B in the placebo arm; blue indicates downregulated pathways. Selected immune pathways are solid dots and labelled when significant (FDR *q*-value < 0.05).

d) GSEA of the differential expression analysis of week 52 kidney samples, comparing individuals who experienced rebound in peripheral blood transcriptomic signatures versus

those who did not, using an immune complex (IC)-stimulated macrophage signature[S3]. The position of the dot indicates the normalized enrichment score (NES). *P*-value is indicated.

***Supplementary Figure S3*: Weighted gene co-expression network analysis (WGCNA) of peripheral blood samples.** Peripheral blood samples were taken at week 0, week 12 and week 52 in a clazakizumab in late AMR study and underwent RNA sequencing (RNA-seq).

a) Heatmap plot of the adjacencies in the eigengene network. Each row and column corresponds to one module eigengene (labelled by colour). In the heatmap, blue indicates low adjacency (negative correlation), while red represents high adjacency (positive correlation).

b) STRING analysis of leading edge genes of the GSEA of T follicular helper (Tfh) cell signatures in the Lightyellow module. Genes were ranked by module membership. Only connected nodes are displayed. Edges represent confidence score >0.4 between genes; thicker line = stronger connection.

***Supplementary Figure S4:* Weighted gene co-expression network analysis (WGCNA) of kidney biopsy samples.** Kidney biopsies were taken alongside peripheral blood samples at week 0, week 11 and week 51 in a clazakizumab in late AMR study and analyzed by microarray.

a) Scatterplot showing 2 module preservation statistics (left, medianRank; right, Zsummary) of identified kidney modules in blood, labelled by colour. When modules were over 1000 genes in size, random sampling was used to select 1000 genes.

b) Heatmap showing enrichment of hallmarks pathways in selected modules identified with WGCNA. Enrichment scores were calculated using single-sample gene set enrichment analysis (ssGSEA) and scaled by row. Red indicates positive enrichment; blue indicates negative.

c) Heatmap of top 20 leading edge genes for the GSEA of ‘*adaptive proximal tubule*’ in the cyan module, in individuals in the clazakizumab arm. Samples (columns) have been classified by the sample time point. Expression data has been normalized and scaled by row (colour represents Z-score).

d) Scatterplot indicating donor-derived cell-free DNA (dd-cfDNA), as a proportion of baseline, at 3 time points in the clazakizumab in late antibody-mediated rejection study. Lines connect samples belonging to individuals. Blue indicates samples belonging to individuals in the placebo arm; red indicates samples belonging to those in the clazakizumab arm.

Supplementary Figure S1

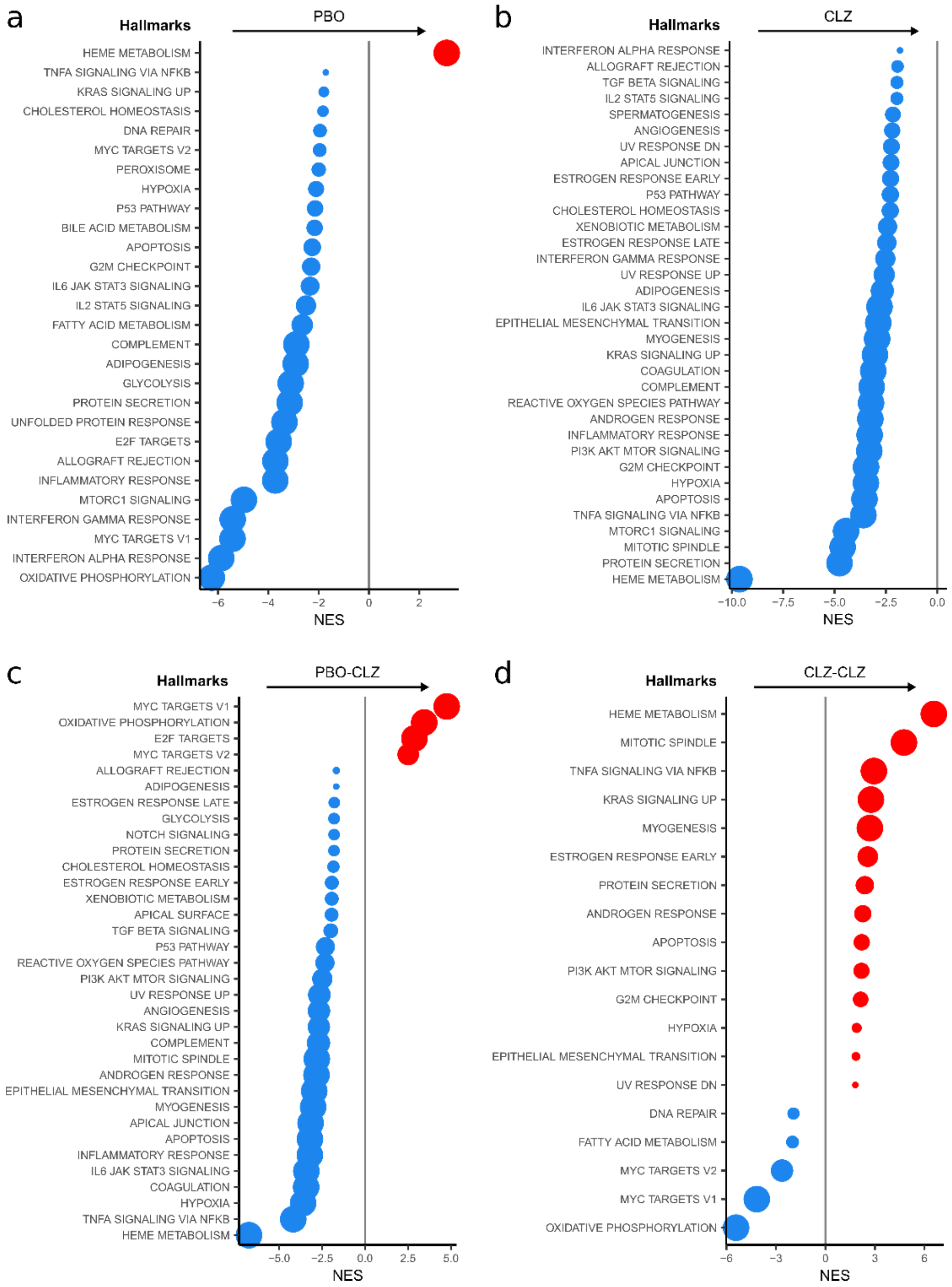

Supplementary Figure S2

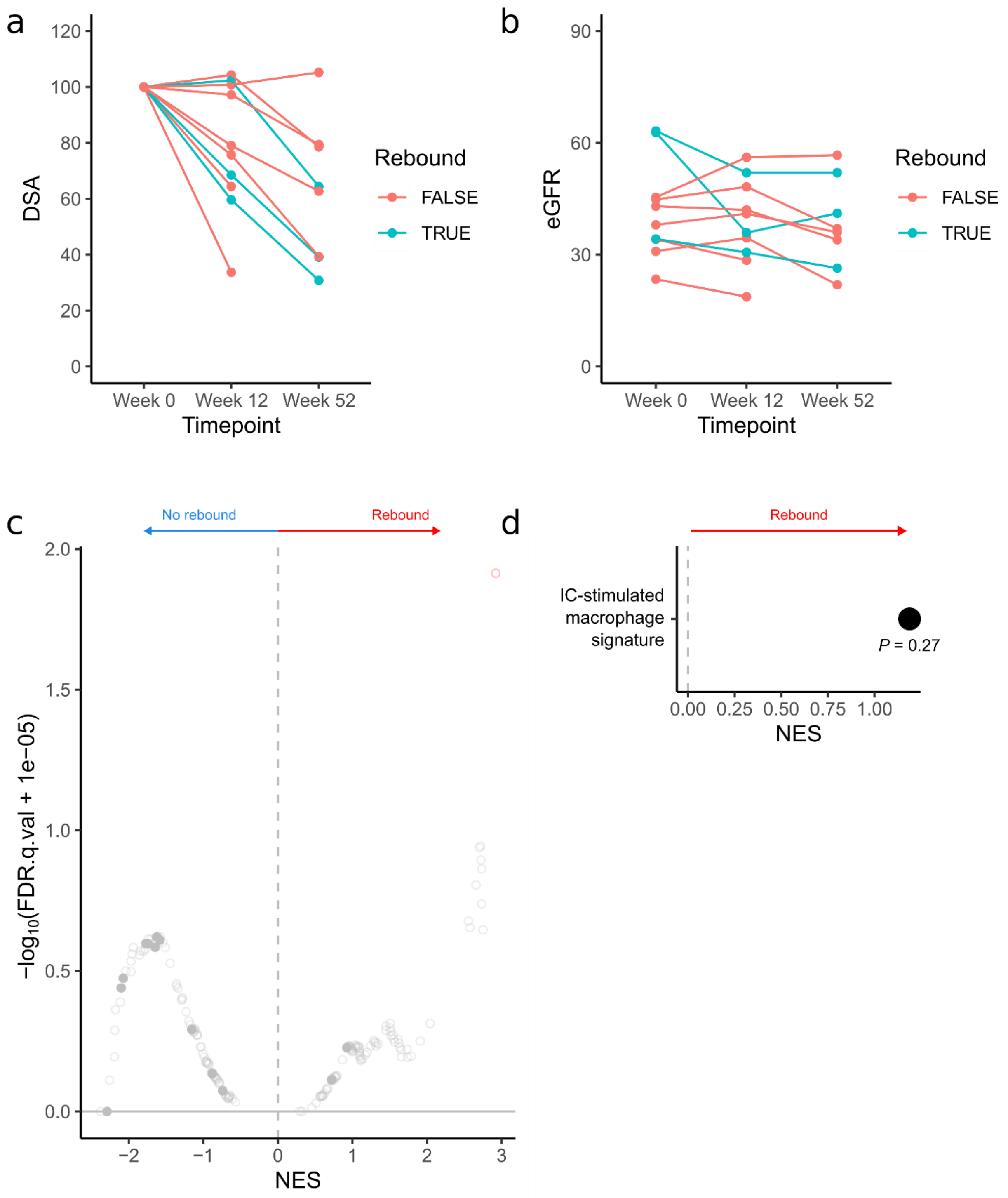

Supplementary Figure S3

a

PBMC modules

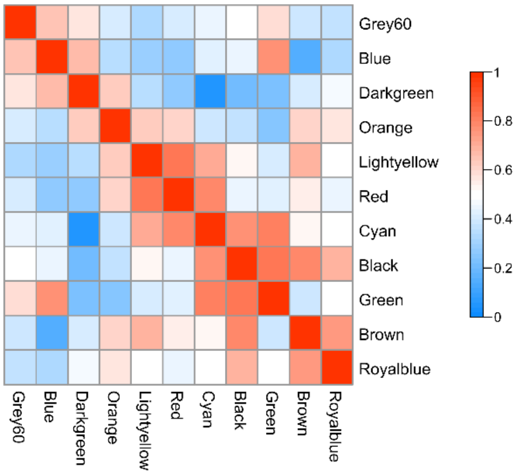

b

Lightyellow Tfh leading edge genes

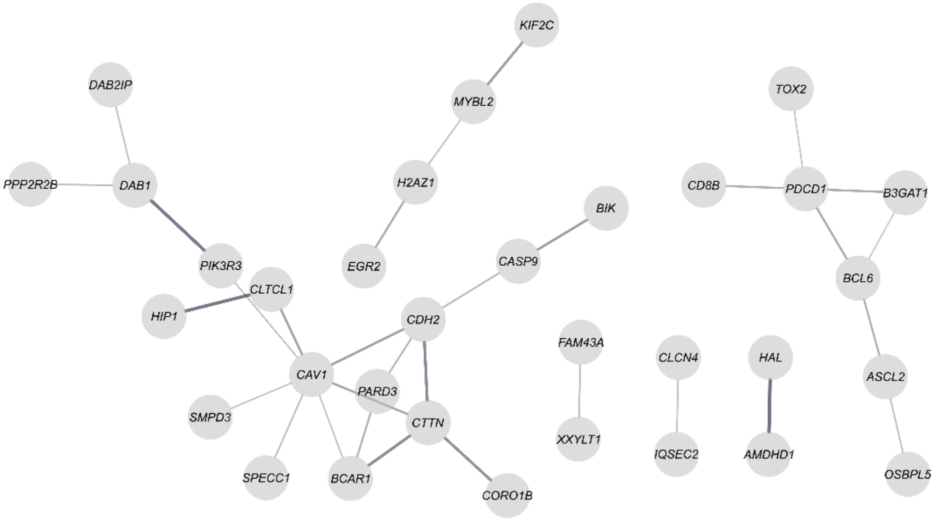

Supplementary Figure S4

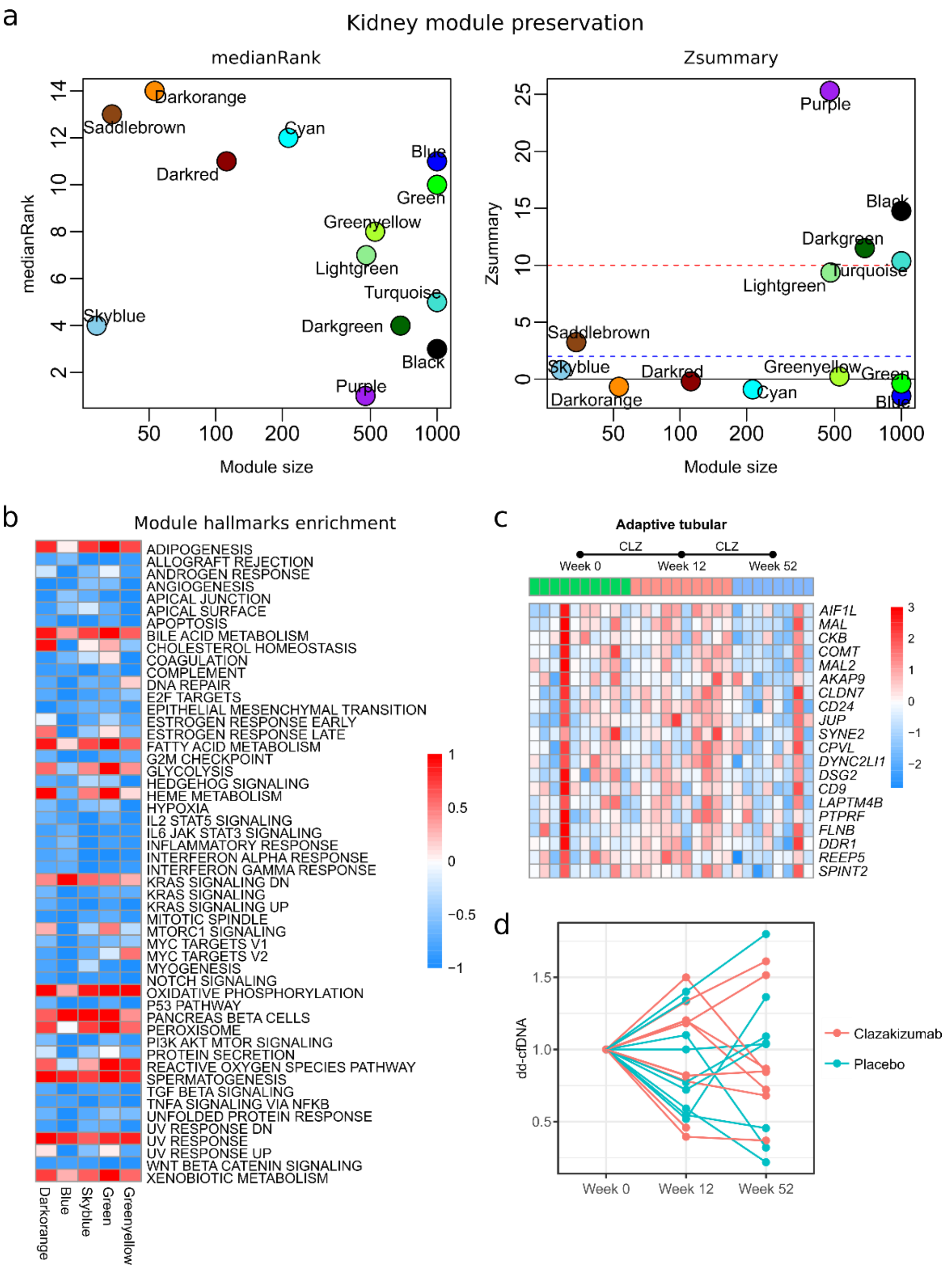

Supplementary References

S1. Kanehisa M, Sato Y, Kawashima M, et al. KEGG as a reference resource for gene and protein annotation. *Nucleic Acids Research*. 2016;44(D1):D457-D462.

S2. Liberzon A, Birger C, Thorvaldsdóttir H, et al. The Molecular Signatures Database (MSigDB) hallmark gene set collection. *Cell Syst*. 2015;1(6):417-425.

S3. Banham GD, Lee CYC, Ferdinand JR, et al. Bromodomain Inhibitors Modulate FcγR-Mediated Mononuclear Phagocyte Activation and Chemotaxis. *Frontiers in Immunology*. 2022;13.

S4. Hao Y, Hao S, Andersen-Nissen E, et al. Integrated analysis of multimodal single-cell data. *Cell*. 2021;184(13):3573-3587.

S5. Stewart BJ, Ferdinand JR, Young MD, et al. Spatiotemporal immune zonation of the human kidney. *Science*. 2019;365(6460):1461-1466.

S6. Weinstein JS, Lezon-Geyda K, Maksimova Y, et al. Global transcriptome analysis and enhancer landscape of human primary T follicular helper and T effector lymphocytes. *Blood*. 2014;124(25):3719-3729.

S7. Xie X, Shi Q, Wu P, et al. Single-cell transcriptome profiling reveals neutrophil heterogeneity in homeostasis and infection. *Nat Immunol*. 2020;21(9):1119-1133.

S8. Lake BB, Menon R, Winfree S, et al. An atlas of healthy and injured cell states and niches in the human kidney. *Nature*. 2023;619(7970):585-594.

S9. Zhang X, Song B, Carlino MJ, et al. An immunophenotype-coupled transcriptomic atlas of human hematopoietic progenitors. *Nat Immunol*. 2024;25(4):703-715.

S10. Langfelder P, Luo R, Oldham MC, et al. Is My Network Module Preserved and Reproducible? *PLOS Computational Biology*. 2011;7(1):e1001057.

S11. Halloran PF, Reeve J, Akalin E, et al. Real Time Central Assessment of Kidney Transplant Indication Biopsies by Microarrays: The INTERCOMEX Study. *Am J Transplant*. 2017;17(11):2851-2862.

S12. Reeve J, Böhmig GA, Eskandary F, et al. Assessing rejection-related disease in kidney transplant biopsies based on archetypal analysis of molecular phenotypes. *JCI Insight*. 2(12):e94197.

S13. Haas M, Loupy A, Lefaucheur C, et al. The Banff 2017 Kidney Meeting Report: Revised diagnostic criteria for chronic active T cell-mediated rejection, antibody-mediated rejection, and prospects for integrative endpoints for next-generation clinical trials. *Am J Transplant*. 2018;18(2):293-307.

S14. Alexa A, Rahnenführer J, Lengauer T. Improved scoring of functional groups from gene expression data by decorrelating GO graph structure. *Bioinformatics*. 2006;22(13):1600-1607.
